# Preparation of TiO_2_-modified Biochar and its Characteristics of Photo-catalysis Degradation for Enrofloxacin

**DOI:** 10.1038/s41598-020-62791-5

**Published:** 2020-04-20

**Authors:** Wen Wang, Jing Zhang, Tianya Chen, Jing Sun, Xiulan Ma, Yujun Wang, Jihong Wang, Zhonglei Xie

**Affiliations:** 1 College of Resources and Environment, Jilin Agricultural University, Changchun, Jilin Province, 130118 People’s Republic of China; 2 College of Plant Science, Jilin University, Changchun, Jilin Province, 130062 People’s Republic of China; 3 College of Construction Engineering, Changchun Sci-Tech University, Changchun, Jilin Province 130600 People’s Republic of China

**Keywords:** Environmental sciences, Materials science

## Abstract

In order to solve the problem that the traditional biochar(BC) has insufficient removal ability of enrofloxacin and TiO_2_ is difficult to recycle. In this study, TiO_2_-modified biochar composites were prepared by impregnation method. Through characterization analysis, The BET specific surface area results indicated that after loading TiO_2_, the specific surface area of TiO_2_-biochar(Ti-BC), TiO_2_-ironized biochar(Ti-FBC) and TiO_2_-alkaline biochar(Ti-KBC) increased by 4.34, 10.43 and 11.52 times, respectively. The analysis results of SEM, EDS, FT-IR, XRD and XPS showed that TiO_2_ was supported on biochar in the anatase state. The UV-vis DRS measurement showed that the band width of Ti-KBC was the smallest and the best catalytic activity. Under 15 W UV lamp (254 nm) irradiation, the photocatalytic degradation process of enrofloxacin by different biochar accords with the first-order kinetic equation. Ti-KBC showed best degradation effect under different initial concentrations of enrofloxacin. When the pH of the solution was 5.0 and the dosage of Ti-KBC was at 2.5 g·L^−1^, the enrofloxacin degradation rate of 100 mg·L^−1^ reached 85.25%. The quenching test confirmed that the active substance O_2_•^—^ played a major role in the photocatalytic degradation process. After five cycles of the test, the degradation rate of Ti-KBC for enrofloxacin was 77.14%, which was still better than that of BC, Ti-BC and Ti-FBC.

## Introduction

Enrofloxacin is the third-generation fluoroquinolone antibiotic which was synthesized in 1987 and is widely used in livestock and aquaculture as antibacterial drugs, because of its highly efficient against mycoplasma^1^. The excessive use of enrofloxacin has also been resulted in a series of pollution problems and there have been many reports on the excessive detection of enrofloxacin in the surface water near the farm^2^. The study found that after the enrofloxacin solution was administered to pigs, the amount of enrofloxacin in the excreta reached 34.44–78.42 mg·kg^−1^ ^3^ Enrofloxacin stays in the natural environment for a long time and produces “super bacteria”, which poses a potential threat to human health^4^. Therefore, it is of great practical significance to explore ways to remove enrofloxacin remaining in the environment.

Biochar is a kind of solid matter which is formed by pyrolysis of biochar raw materials such as corn stover and wood chips under oxygen-limited conditions, and has strong adsorption capacity for pollutants^5^. Because of its rich surface functional groups and large specific surface area^6^, and the advantages of easy availability of raw materials and low price, the use of biochar as a new type of adsorbent has been increasing in recent years^7,8^. Compared with traditional activated carbon, biochar has limited adsorption capacity for enrofloxacin in the environment. In order to enhance the adsorption capacity of biochar, the biochar is usually modified by physical and chemical methods such as acid-base and metal solution impregnation^9,10^.

TiO_2_ is widely used in photocatalysis research due to its good catalytic performance and non-toxic and reusable properties^11^. However, the traditional nano-TiO_2_ catalytic technology has the disadvantages of easy to be inactivated and difficult to recycle, which limits its application prospects in actual production. In order to improve the catalytic efficiency of TiO_2_, its often modified by ion doping^12^, immobilization and other techniques. Du *et al*.^13^ carried out TiO_2_ on carbon nanotubes by sol-gel method and founded that the degradation rate of methylene blue by TiO_2_ loaded with 2% carbon nanotubes was enhanced to 90.6%. Radek Zouzelka *et al*.^14^ synthesized the carbon nanotube TiO_2_ porous film, and the degradation effect of the composite on 4-chlorophenol reached 96% after 0.6% carbon nanotubes supported TiO_2_. Yang *et al*.^15^. Modified activated carbon fiber (ACF) with HNO_3_ and synthesized a TiO_2_/ACF composite, the degradation efficiency of the composite to methylene blue reached 99.99% after 30 minutes of reaction.

Previous research work has focused on the choice of carriers for immobilized TiO_2_, but there have been few reports on carrier studies before loading. In this study, biochar was prepared with corn stover, and then biochar modified with iron(FeCl_3_) and alkali(KOH) were prepared by impregnationt and TiO_2_/modified biochar composite was prepared by impregnation calcination method. Through the structural characterization of the composite and the photocatalytic degradation test, the degradation mechanism of enrofloxacin was clarified.

## Results

### Physical and chemical properties of biochar

Table 1 shows the physical and chemical properties of BC, Ti-BC, Ti-FBC and Ti-KBC. The surface acid group content of the four composites was determined by Boehm titration. The surface of BC was mainly composed of a basic group, and the basic group of Ti-BC loaded with TiO_2_ was slightly increased. The content of basic groups on the surface of the prepared Ti-FBC composites decreased obviously, and the content of acidic groups increased by 2.40 times. The surface of the prepared Ti-KBC composites decreased, and the content of basic groups increased by 1.49 times. The difference in acid-base group content was one of the reasons for the difference in the ability of biochar to remove pollutants^16^. The result from BET specific surface area measurement showed that the specific surface areas of the biochar Ti-BC, Ti-FBC and Ti-KBC were 15.24, 36.65 and 40.46 m^2^·g^−1^, which increased by 4.34, 10.43 and 11.52 times compared with BC, respectively. The micropore volume of Ti-FBC and Ti-KBC accounted for 66.47% and 74.44% of the total pore volume, respectively. The surface structure and micropore content of biochar were also one of the reasons that affecting its adsorption performance^17^.

**Table 1:** Physicochemical properties of BC, Ti-BC,Ti-FBC and Ti-KBC.

Sample	Acidic group/nmol·g^−1^	Basic group/nmol·g^−1^	BET surface area/m^2^·g^−1^	Total pore volume/cm^3^·g^−1^	Micropore volume/cm^3^·g^−1^	Average pore width/nm
BC	0.2473	0.6399	3.5124	0.0129	0.0043	1.2940
Ti-BC	0.2437	0.6492	15.2385	0.0388	0.0130	1.1397
Ti-FBC	0.5935	0.2391	36.6511	0.2535	0.1685	0.8578
Ti-KBC	0.2012	0.9536	40.4570	0.3013	0.2243	0.6192

### Scanning electron microscopy and EDS of biochar

**Figure 1:**
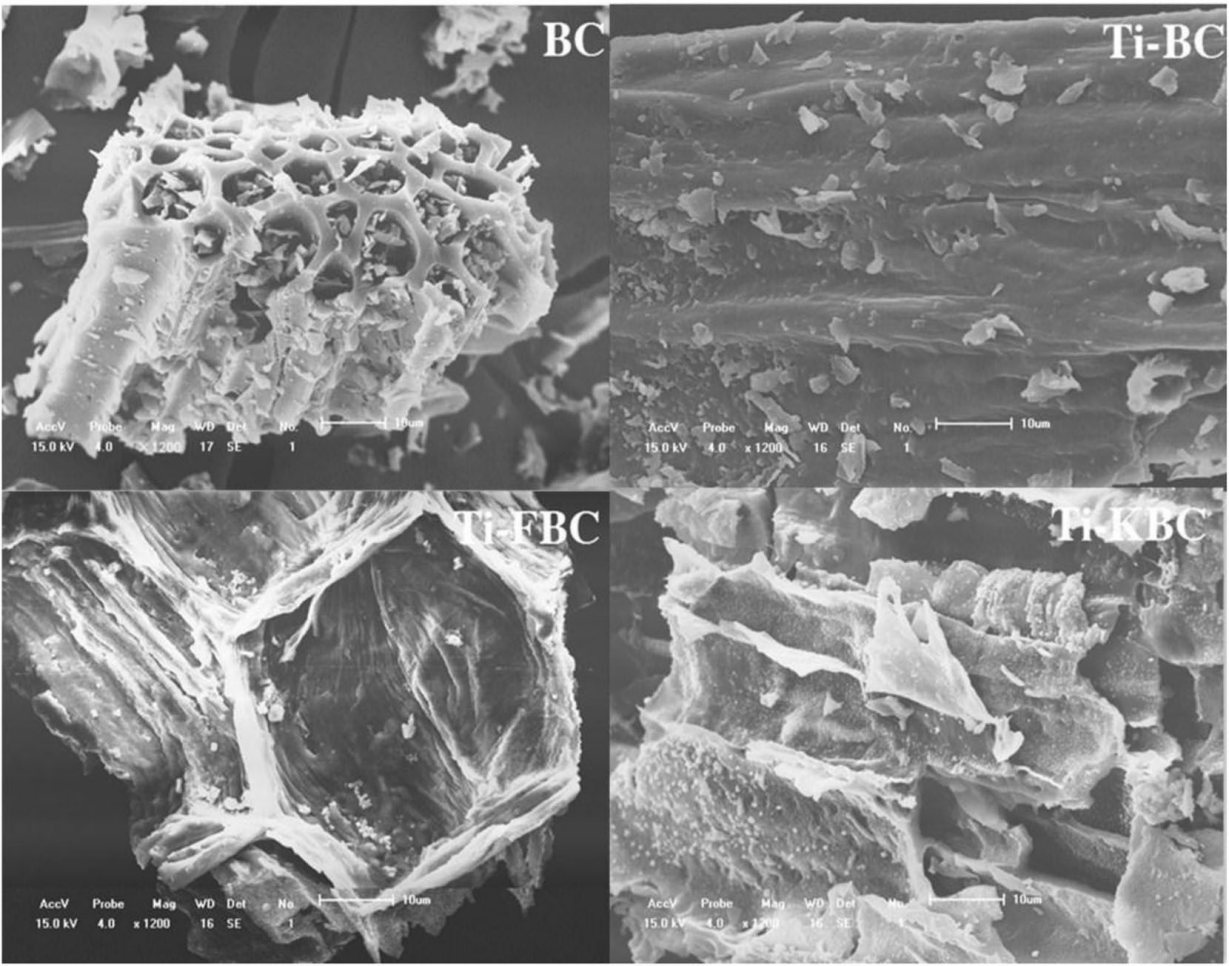
Scanning electron micrograph of BC, Ti-BC, Ti-FBC and Ti-KBC (1200 times).

**Figure 2:**
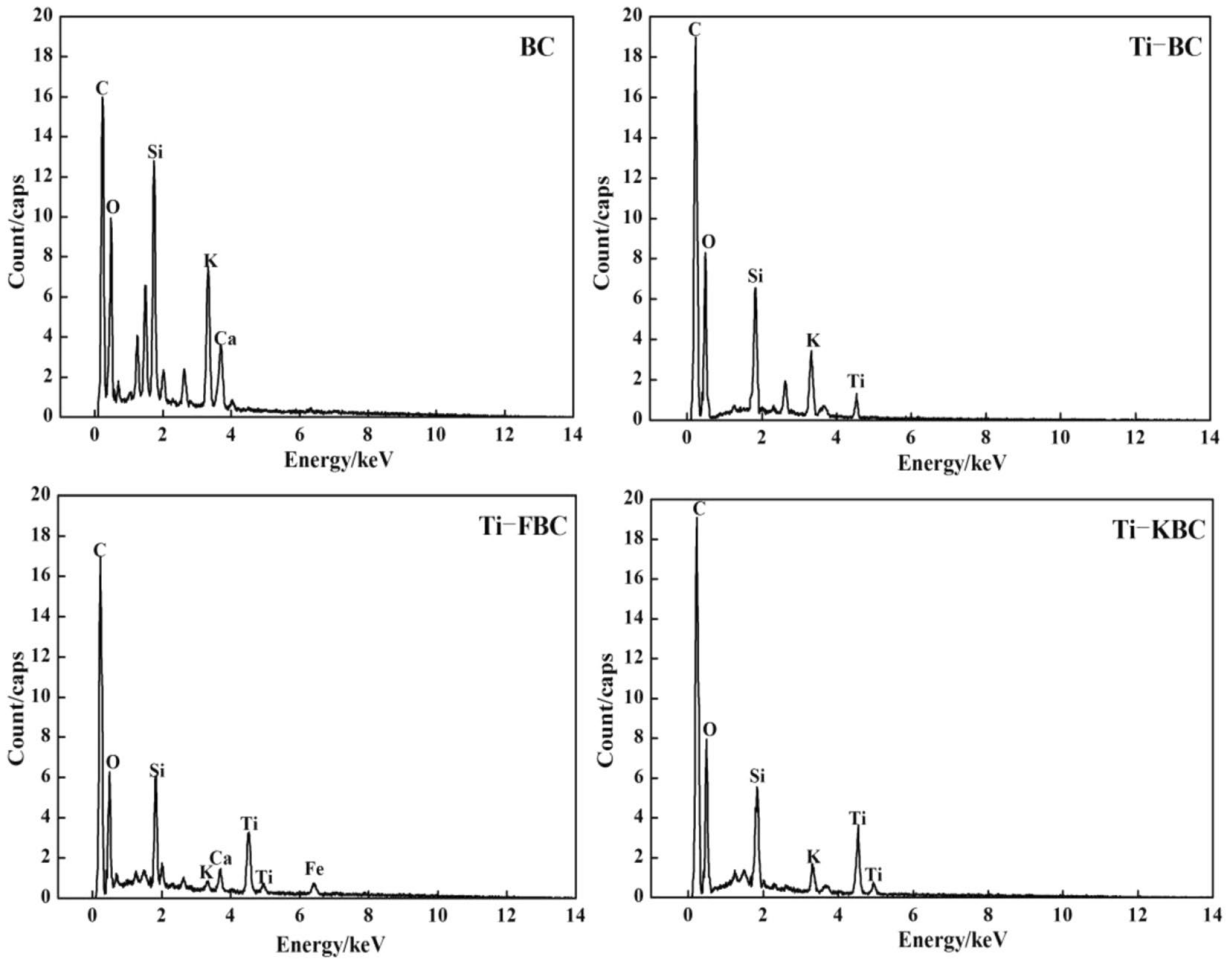
Energy spectra of BC, Ti-BC, Ti-FBC and Ti-KBC.

Figure 1 shows an SEM image of BC, Ti-BC, Ti-FBC and Ti-KBC in which the surface structure of the four carbons was evidently different. The BC has a small amount of impurity particles are attached to the surface, and the pores are occupied by a large number of debris particles. After the high temperature pyrolysis, the inner wall of the biochar collapses, and the fallen debris particles stay inside the pores, causing blockage. Compared with the SEM of BC, there were particulate matter in the carbon surface and pores of Ti-BC, Ti-FBC and Ti-KBC. Figure 2 shows EDS spectra of the four carbon. Ti-BC, Ti-FBC and Ti-KBC has Ti detected in the EDS spectra, which was not found in BC. Therefore, it was judged that the particulate matter appearing in the composite material in Fig. 1 was TiO_2_. The EDS spectrum results showed that the AT(%) of Ti in Ti-BC, Ti-FBC and Ti-KBC are 0.409, 1.410, and 2.142, respectively. It can also be seen from Fig. 2 that after iron and alkali modification, the response values of Ti in the EDS spectra of Ti-FBC and Ti-KBC were stronger than that of Ti-BC, which indicating that the modified biochar can support more TiO_2_ under the same loading conditions.

### Infrared spectroscopy of biochar

**Figure 3:**
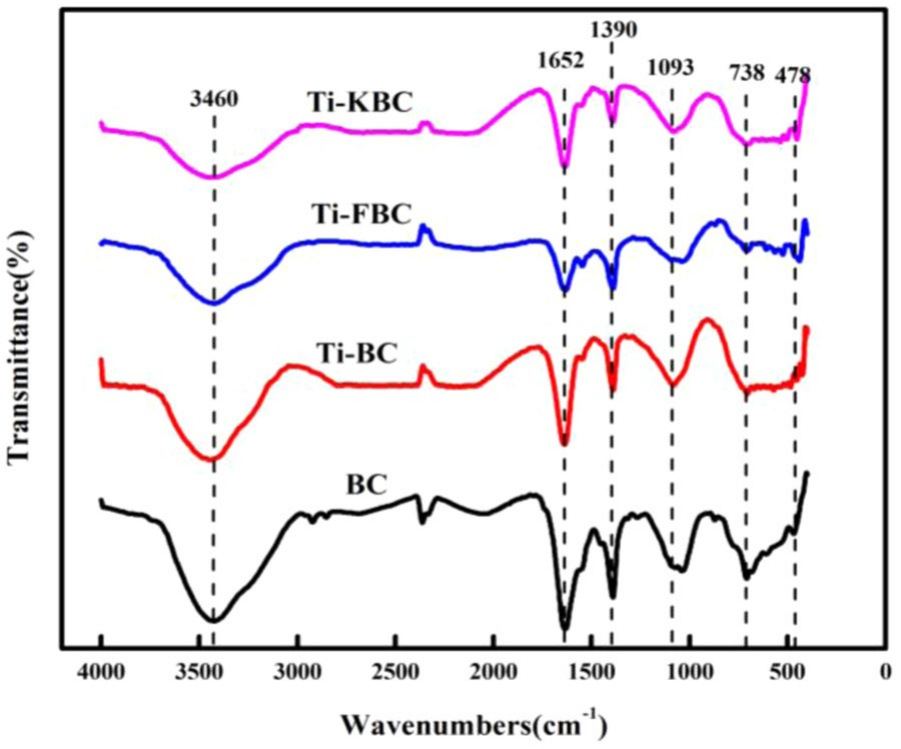
Infrared spectra of BC, Ti-BC, Ti-FBC and Ti-KBC.

Figure 3 shows the infrared spectrum of BC, Ti-BC, Ti-FBC and Ti-KBC. The characteristic peaks of BC, Ti-BC, Ti-FBC and Ti-KBC at 3423 cm^−1^ should be –OH stretching vibration peaks^18^. In the prepared composite material, the peak intensity here was obviously weakened, indicated that after the biochar was loaded with TiO_2_, the hydroxyl group on the surface of the biochar undergoes a fracture association reaction with the TiO_2_, and thus the surface hydroxyl group content was lowered. The characteristic peak at 1652 cm^−1^ was C=O bond stretching vibration. The four kinds of biochar have strong absorption peaks here, indicated that the surface of the biochar before and after loading contains carbonyl compounds such as carboxylic acid^19^. The peak at 1390 cm^−1^ was the in-plane bending vibration of –CH_3_, which was a characteristic vibration peak of charcoal matter; the symmetric stretching vibration absorption peak of C–O–C bond at 1093 cm^−1 19^. The absorption band at 478–738 cm^−1^ in Fig. 2 was due to the stretching vibration of the Ti–O–Ti bond and the Ti-O bond in the TiO_2_ molecule^13^. This characteristic peak appears only in the infrared spectrum of Ti-BC, Ti-FBC and Ti-KBC and is not found in BC, which indicated that TiO_2_ has been supported on the surface of the modified biochar.

### XRD of biochar

**Figure 4:**
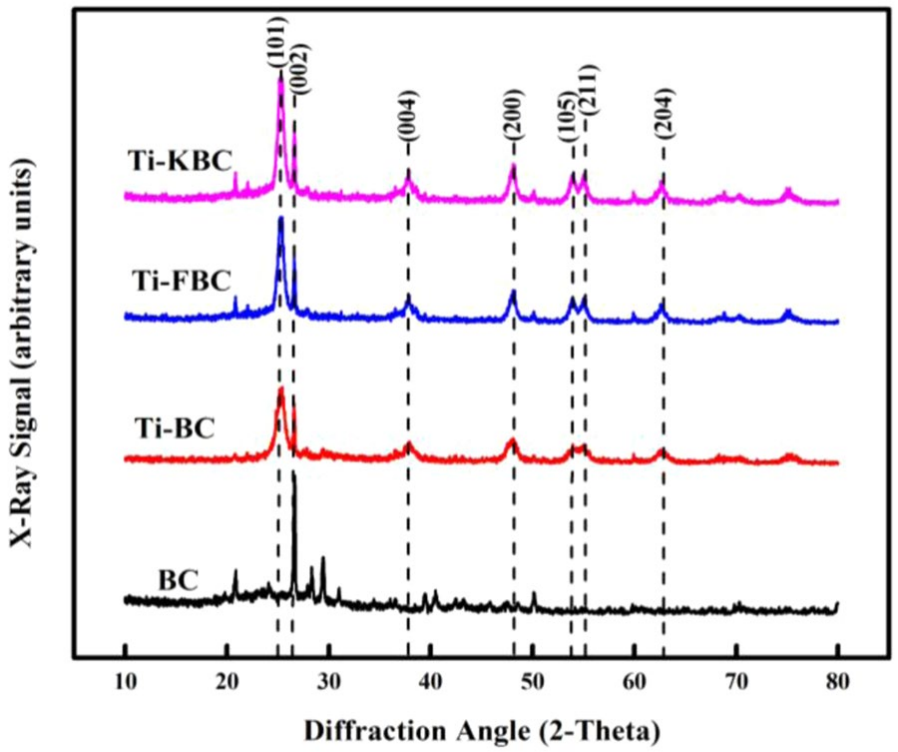
X-ray diffraction patterns of BC, Ti-BC, Ti-FBC and Ti-KBC.

Figure 4 shows the XRD patterns of BC, Ti-BC, Ti-FBC and Ti-KBC. The four biochar have obvious diffraction peaks at 2θ = 26.6°, and the graphite microcrystal diffraction peak d_002_^20^ was determined according to the analysis result of Jade. After the corn stalk biochar was modified and loaded, the diffraction peak intensity was weakened at 2θ = 26.6°, and the crystallinity decreased. The surface defects and porosity of the biochar increased, so the specific surface area of the composite material also increased. From the XRD patterns of Ti-BC, Ti-FBC and Ti-KBC in Fig. 4, the diffraction peaks located at 25.26°, 37.88°, 48.08°, 53.94°, 55.00° and 62.56° were corresponded to (101), (004), (200), (105), (211) and (204) crystal planes of anatase TiO_2_ (ICSD: 01–089). The results revealed that TiO_2_ supported on each biochar material existed in an anatase state under high temperature calcination, this was consistent with the analysis results of the EDS spectra.

### XPS of biochar

**Figure 5:**
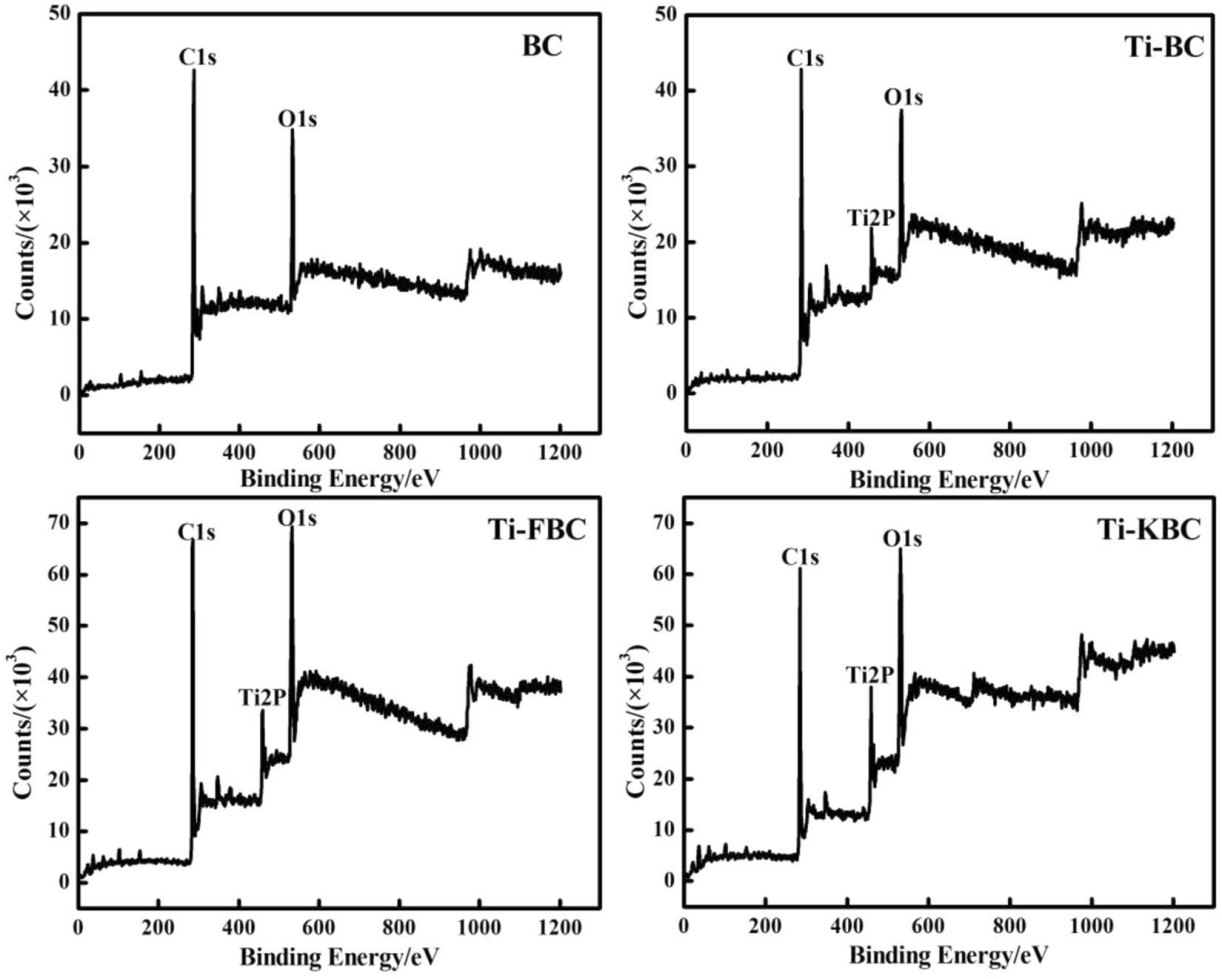
XPS spectra of BC, Ti-BC, Ti-FBC and Ti-KBC.

**Figure 6:**
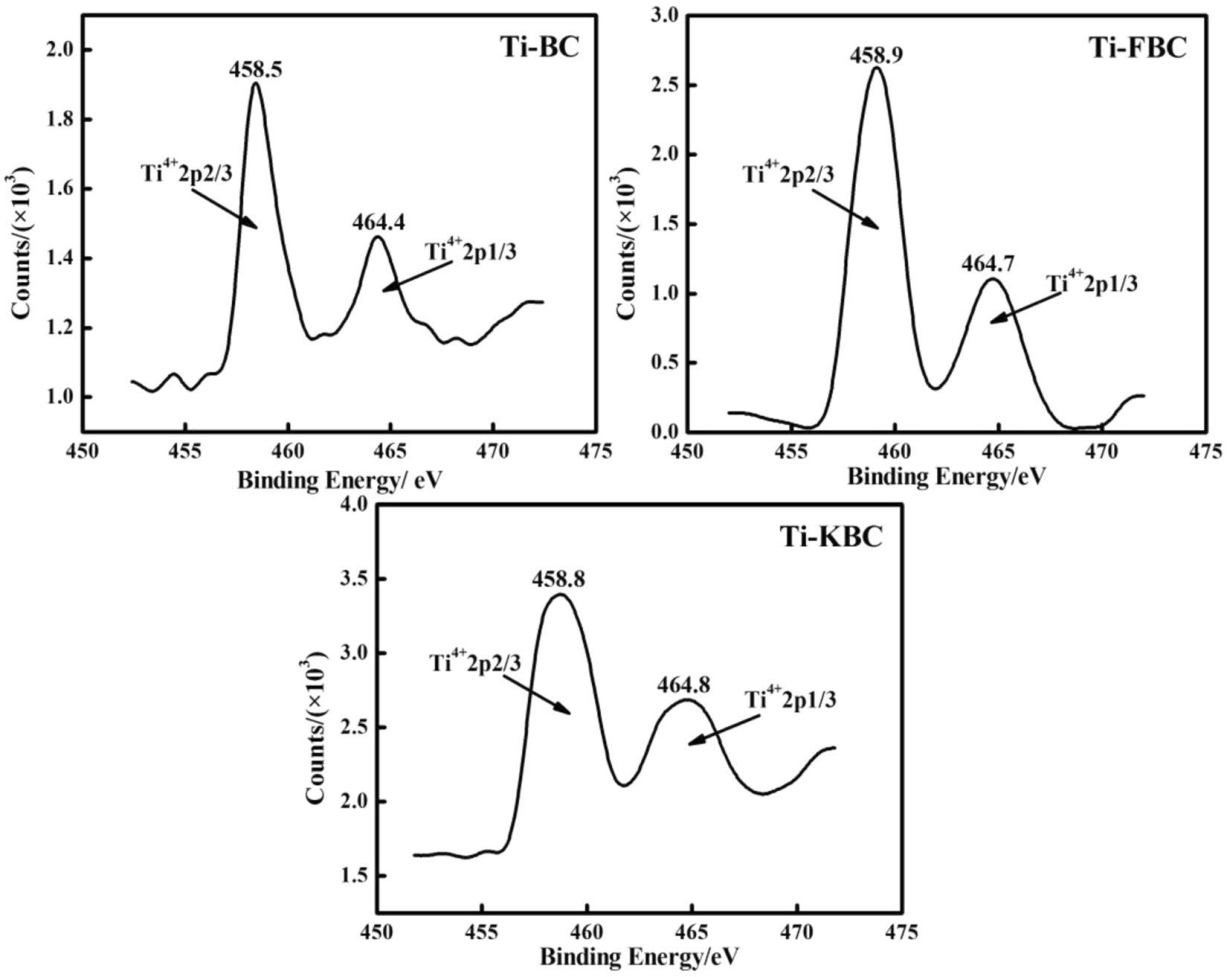
Ti2p peaks of Ti-BC, Ti-FBC and Ti-KBC.

Figure 5 shows the XPS spectra of BC, Ti-BC, Ti-FBC and Ti-KBC. From Fig. 5, it can be seen that the C1s and O1s lines appear in the XPS spectra of the four biochar. Compared with BC, Ti2p lines appear in the spectra of Ti-BC, Ti-FBC and Ti-KBC, confirming the presence of Ti element in the composite, which was consistent with the previous characterization results of EDS, XRD, etc. In order to determine the existence form of Ti element, the lines near the Ti2p peak in Ti-BC, Ti-FBC and Ti-KBC are enlarged to obtain the fine spectrum of Ti2p, as shown in Fig. 6. It can be seen from Fig. 6 that the Ti2p energy levels of Ti-BC, Ti-FBC and Ti-KBC are split into two energy levels due to the spin-orbit coupling of the electrons. Peak fitting was performed based on the contribution of different titanium oxides to Ti2p. The peaks at 458.5, 458.9, and 458.8 eV corresponded to the peaks of Ti2p3/2, and the peaks at 464.4, 464.7, and 464.8 eV corresponded to Ti2p1/2 peaks. The actual measurement results are close to the theoretical values Ti2p3/2 (458.8 eV) and Ti2p1/2 (462.2 eV), indicated that the Ti valence state in Ti-BC, Ti-FBC and Ti-KBC is Ti^4+^ and exists as TiO_2_^21^.

### UV-vis DRS of biochar

**Figure 7:**
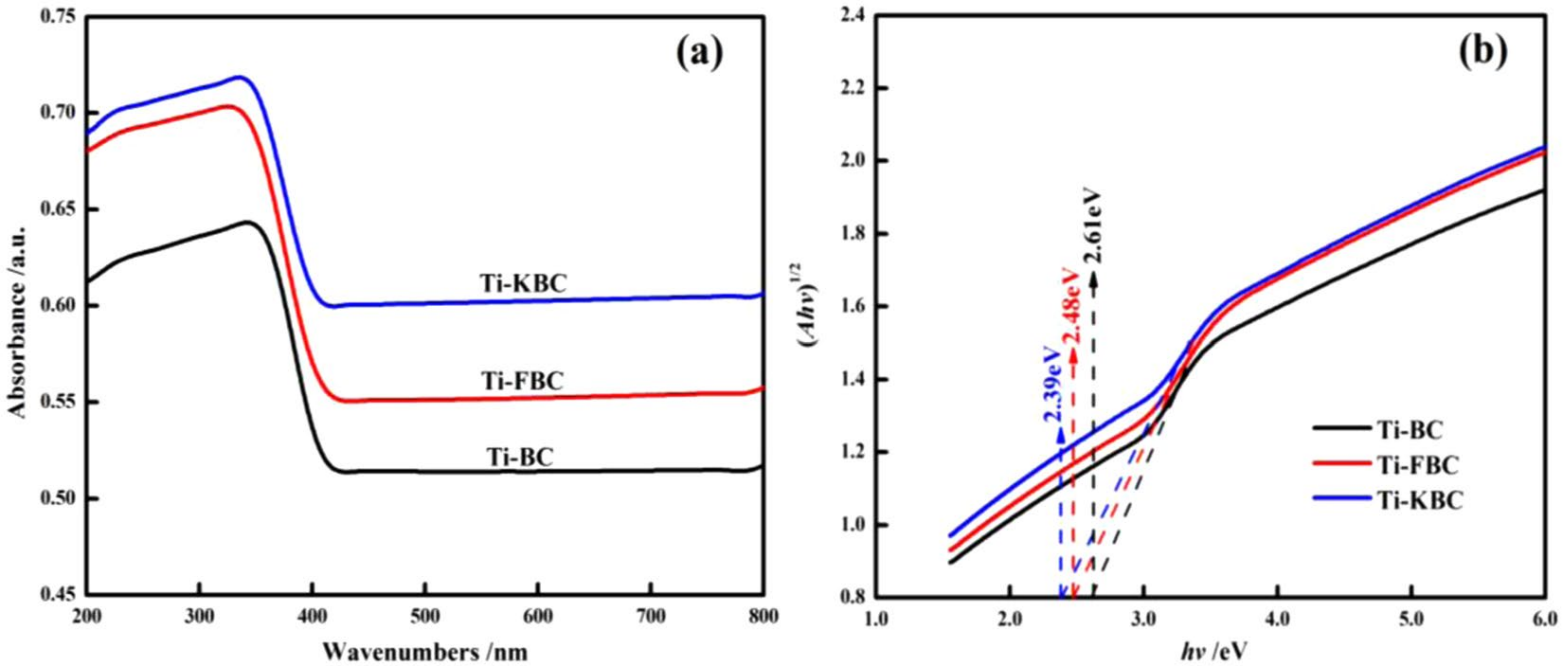
The UV-vis DRS adsorption spectra (**a**) and the band gap (**b**) of Ti-BC, Ti-FBC and Ti-KBC.

Figure 7(a,b) are the UV-vis DRS spectra and band gap of Ti-BC, Ti-FBC and Ti-KBC. It can be seen from Fig. 7(a) that the composite material after TiO_2_ loading has strong absorption in the ultraviolet region. Compared with Ti-BC, the maximum light absorption edges of Ti-FBC and Ti-KBC are red-shifted and show higher light absorption in the visible light region. In order to further explore the optical properties of biochar, the band gap *E*_*g*_ of Ti-BC, Ti-FBC and Ti-KBC was calculated by Tauc plot Eq. (1)^22^. As shown in Fig. 7(b), according to the Eq. (1), the forbidden band widths of Ti-BC, Ti-FBC and Ti-KBC were calculated to be 2.69 eV, 2.52 eV, and 2.41 eV, respectively.

1 $${(\alpha hv)}^{1/n}=A({h}_{v}-{E}_{g})$$

Where *α* is the absorption coefficient, and Abs value is used in this article; h is the Planck constant, 6.626 × 10^–34^ J·s; *ν* is the frequency, *hν* = *hc/λ*, *c* is the speed of light, 3 × 10^8^ m·s^−1^, *λ* is the wavelength of light, m; TiO_2_ is an indirect bandgap semiconductor, *n* = 2; *A* is a constant; *Eg* is the band gap, eV. Plot *hv* as the abscissa (αhv)^1/2^ as the ordinate, and then tangent to get *Eg*.

### Photodegradation kinetics test

**Figure 8:**
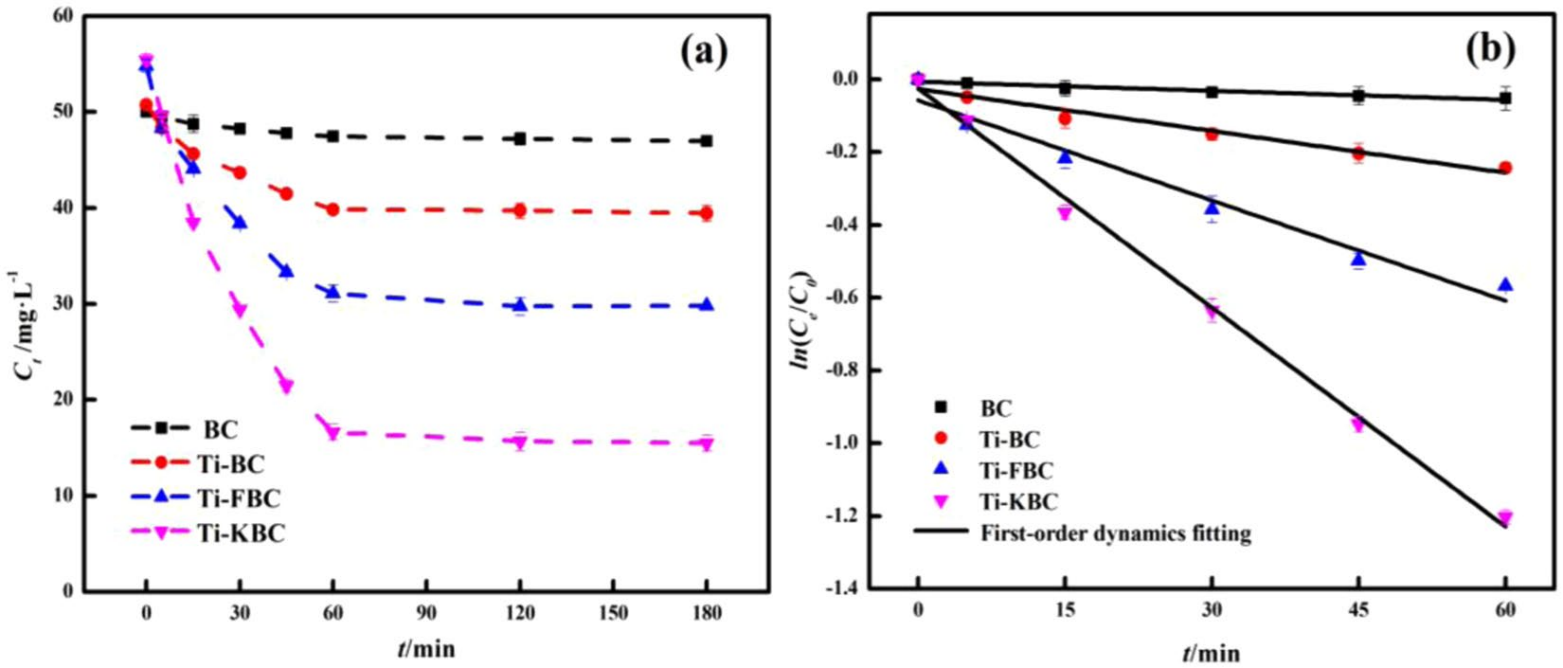
Photocatalytic kinetic curves(**a**) and fitting curves(**b**) of enrofloxacin by BC, Ti-BC, Ti-FBC and Ti-KBC.

Figure 8(a) shows the curve of the equilibrium concentration of enrofloxacin over time in the BC, Ti-BC, Ti-FBC and Ti-KBC systems under UV light. According to the Fig. 8(a), after the solution system has undergone dark reaction absorption-desorption equilibrium, the equilibrium concentrations of enrofloxacin in the BC, Ti-BC, Ti-FBC, and Ti-KBC systems were 50.00, 50.76, 54.80, and 55.45 mg·L^−1^, respectively. Under 15 W UV lamp (254 nm) irradiation, the equilibrium concentration of enrofloxacin in the solution gradually decreases with the increase of the photochemical reaction time, and the equilibrium concentration of enrofloxacin tended to equilibrium after 60 minutes of the photocatalytic time. After two stages of adsorption-photolysis, the equilibrium concentrations of enrofloxacin in the BC, Ti-BC, Ti-FBC, and Ti-KBC degradation systems were 46.97, 39.43, 29.78, and 15.50 mg·L^−1^, and the degradation rates of enrofloxacin were 53.01%, 60.60%, 70.32%, and 84.65%, respectively, according to Eq. (1).

The kinetic analysis of enrofloxacin photodegradation was calculated according to Eqs. (3), (4). Figure 8(b) was a photocatalytic first-order kinetics fit curve of enrofloxacin on BC, Ti-BC, Ti-FBC and Ti-KBC for 60 min, and Table 2 showed the rate parameters of photocatalytic degradation of enrofloxacin by different biochar and the proportion of adsorption under dark reaction. As can be seen from Table 2, the degradation rates of enrofloxacin by BC, Ti-BC, Ti-FBC and Ti-KBC were 0.0008, 0.0038, 0.0090, and 0.0101 min^−1^, respectively. After loaded TiO_2_, the degradation rates of enrofloxacin by Ti-BC, Ti-FBC and Ti-KBC were increased by 4.75, 11.25, 25.13 times, respectively. The degradation rate of enrofloxacin was significantly improved by the Ti-FBC and Ti-KBC.

2 $$\eta =\frac{{C}_{0}-{C}_{{\rm{t}}}}{{C}_{0}}\times 100 \% $$

3 $$\frac{{\rm{d}}{C}_{t}}{dt}=-\,r{C}_{0}+b$$

Where *C*_*0*,_ and *C*_*t*_ are the concentrations of enrofloxacin in the solution at the initial, and time t, mg·L^−1^; *η* is the degradation rate of enrofloxacin, %; *t* is the reaction time, min; *r* is the first-order power Learning constant, min^−1^; *b* is a constant.

**Table 2:** Rate parameters for catalytic degradation of enrofloxacin by BC, Ti-BC, Ti-FBC and Ti-KBC.

Sample	Kinetic equations	*r* (min^−1^)	*R*^2^
BC	*ln*(*C*_*e*_/*C*_0_) = −0.0008*t* + 0.0065	0.0008	0.9415
Ti-BC	*ln*(*C*_*e*_/*C*_0_) = −0.0038*t* + 0.0260	0.0038	0.9643
Ti-FBC	*ln*(*C*_*e*_/*C*_0_) = −0.0092*t* + 0.0057	0.0092	0.9692
Ti-KBC	*ln*(*C*_*e*_/*C*_0_) = −0.0201*t* + 0.0245	0.0201	0.9970

According to Eq. (3), the integral is:

4 $$\mathrm{ln}\,\frac{{C}_{t}}{{C}_{0}}=-\,rt+b$$

### Degradation effect of enrofloxacin at different initial concentrations

**Figure 9:**
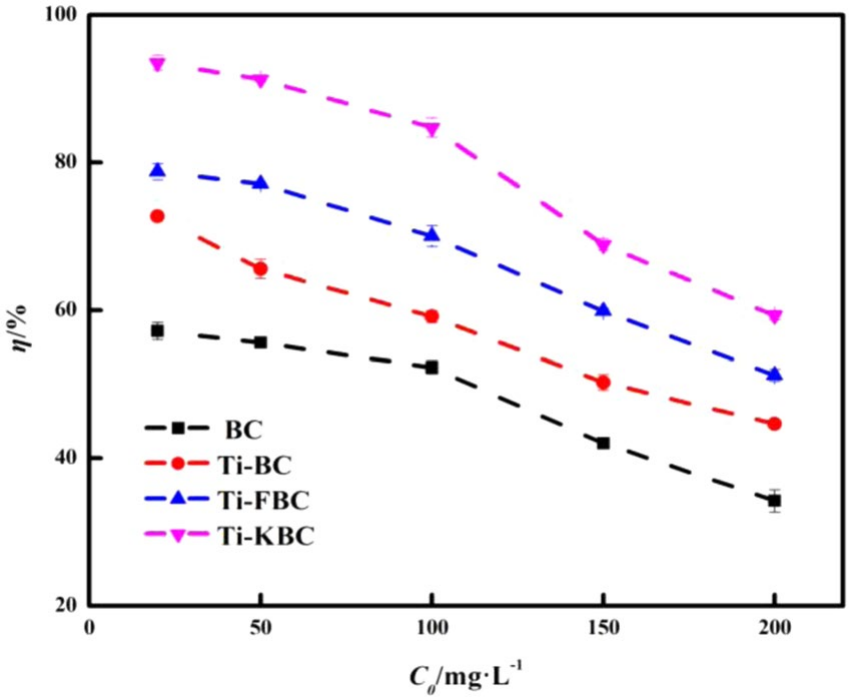
Effects of different initial concentrations on the degradation rate of enrofloxacin.

Figure 9 shows the degradation of enrofloxacin by BC, Ti-BC, Ti-FBC and Ti-KBC at different initial concentrations. When the dosages of BC, Ti-BC, Ti-FBC and Ti-KBC were all 2.5 g·L^−1^, within the test concentration range, the degradation rates of four biochar on enrofloxacin decreased with the increase of the initial concentration. Under different initial concentration conditions, the degradation rates of the four biochar for enrofloxacin were order by Ti-KBC > Ti-FBC > Ti-BC > BC.

### Degradation effect of enrofloxacin at different initial pH values

**Figure 10:**
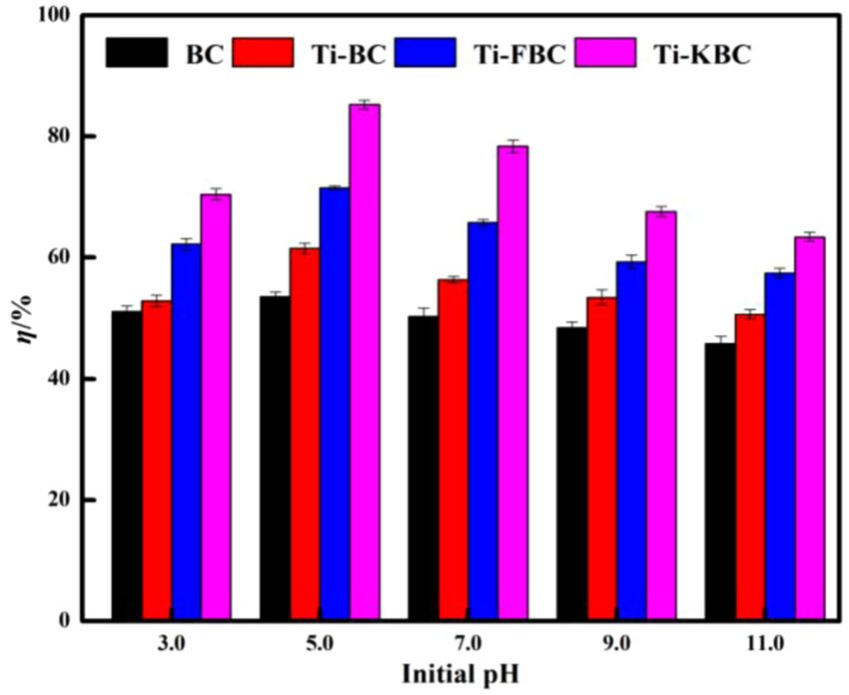
Effect of different initial pH values on the degradation rate of enrofloxacin.

**Figure 11:**
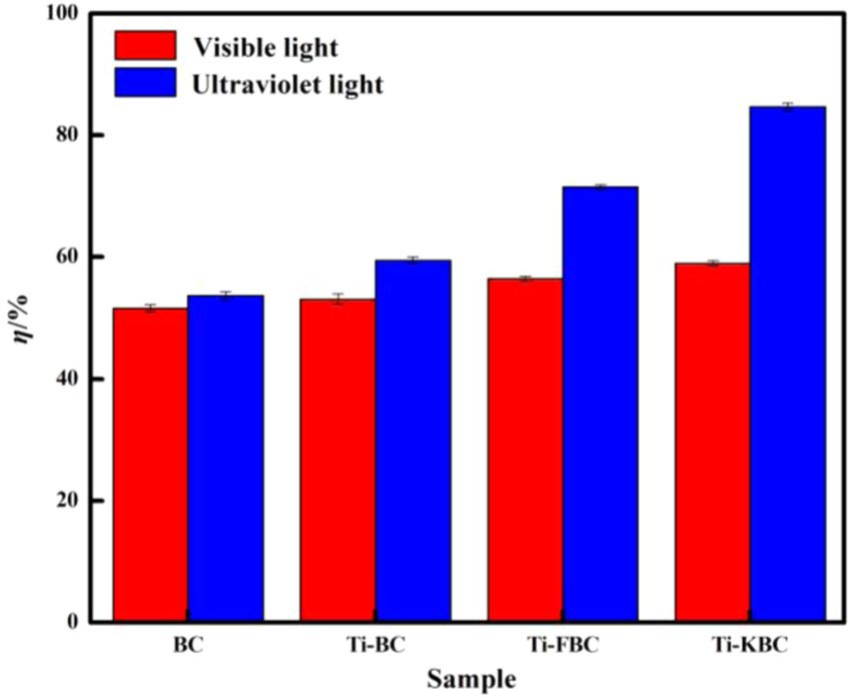
Effects of different light conditions on the degradation rate of enrofloxacin.

Figure 10 shows the changes of BC, Ti-BC, Ti-FBC and Ti-KBC degradation efficiency of enrofloxacin under different initial pH conditions. It can be seen from Fig. 11 that with the increase of the initial pH value of the solution, the degradation rates of the four biochar on enrofloxacin all increase first and then decrease. In different pH values, the degradation rates of the four biochar for enrofloxacin were order by Ti-KBC > Ti-FBC > Ti-BC > BC. When the initial pH = 5.0, BC, Ti-BC, Ti-FBC and Ti-KBC showed the optimum degradation efficiency of enrofloxacin, with the maximum degradation rates of 53.60%, 61.48%, 71.49% and 85.25%, respectively.

### Degradation effect of enrofloxacin by different light sources

Figure 11 shows the degradation effect of BC, Ti-BC, Ti-FBC and Ti-KBC on enrofloxacin under different illumination conditions. After dark reaction adsorption-desorption equilibrium and 60 min of visible light irradiation, the degradation rates of enrofloxacin by BC, Ti-BC, Ti-FBC and Ti-KBC under the irradiation of visible light were 51.58% and 53.11%, 56.41%, 58.97%, respectively. After UV light irradiation for 60 min, the degradation of enrofloxacin by each biochar was 53.60%, 59.48%, 71.49%, and 84.65%, respectively. In summary, the degradation of enrofloxacin by BC, Ti-BC, Ti-FBC and Ti-KBC was better under ultraviolet light irradiation than that under visible light irradiation.

### Identification of photodegradable active substances

**Figure 12:**
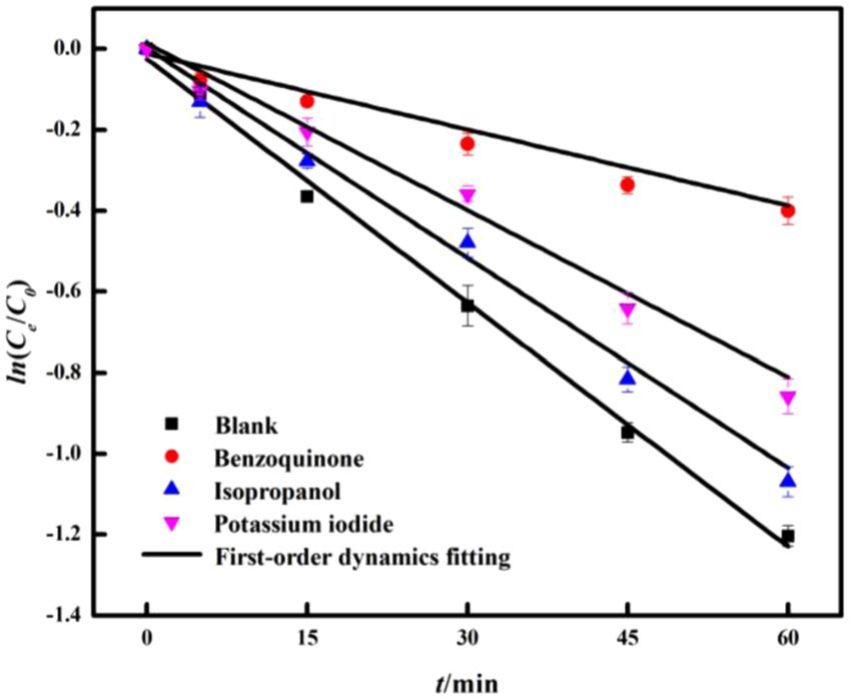
Kinetic fit curve of catalytic degradation of enrofloxacin by different quenchers.

The main active substances of enrofloxacin catalyzed by the supported materials were investigated by the quenching test. Figure 12 shows the kinetic fit curve of the enzymatic degradation of enrofloxacin by adding different quenchers. The specific parameters are shown in Table 3. The active material contribution rate was calculated according to Eq. (5). Table 3 shows that the degradation rate of enrofloxacin was 0.0201 min^−1^ in the unquenched group. After Benzoquinone was added, O_2_•^—^ did not participate in catalytic degradation reaction in the solution. At this time, the degradation rate of enoxacin decreased to 0.0065 min^−1^. According to Eq. (5), the contribution rate of O_2_•^—^ was about 67.66%. In the experimental group with Isopropanol, the degradation rate was 0.0175 min^−1^, and •OH did not participate in the reaction. It was calculated that •OH contributed about 12.94% to catalytic degradation. After adding KI to the solution, the •OH and h^+^ (holes) in the reaction system did not participate in the degradation process. The degradation rate of the test group was 0.0139 min^−1^, and the degradation contribution rate was about 30.85%. By compared and analyzed the contribution rate of different active substances to enrofloxacin degradation, it can be concluded that the main active substance in this reaction system was O_2_•^—^.

5 $$R\approx \frac{r-{r}_{e}}{r}\times 100 \% $$

Where *R* is the contribution rate of different active substances to the degradation of enrofloxacin, %; *r* is the first-order kinetic rate constant of enrofloxacin photodegradation measured by the unquenched test group, min^−1^; *r*_0_ is different The first order kinetic rate parameter measured after the quencher, min^−1^.

**Table 3:** Catalytic degradation rate parameters of enrofloxacin by different quenchers.

Quencher	Kinetic equations	*r* (min^−1^)	*R*^2^
Blank	*ln*(*C*_*e*_/*C*_0_) = −0.0201*t* − 0.0245	0.0201	0.9415
Benzoquinone	*ln*(*C*_*e*_/*C*_0_) = −0.0065*t* − 0.0279	0.0065	0.9643
Isopropanol	*ln*(*C*_*e*_/*C*_0_) = −0.0175*t* − 0.0105	0.0175	0.9692
Potassium iodide	*ln*(*C*_*e*_/*C*_0_) = −0.0139*t* + 0.0029	0.0139	0.9900

### Analysis of cyclic test of TiO_2_-modified biochar

**Figure 13:**
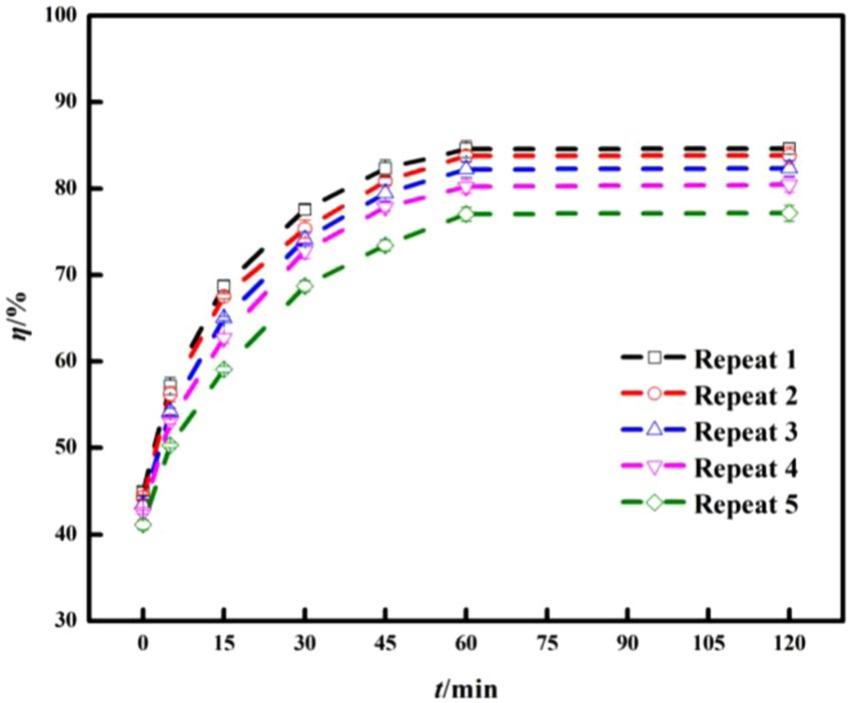
Effect of different cycle times on the degradation efficiency of enrofloxacin.

The photocatalytic test showed that Ti-KBC had the highest degradation rate of enrofloxacin. Therefore, the cycle test was performed with Ti-KBC, and the results were shown in Fig. 13. At the initial concentration of enrofloxacin 100 mg·L^−1^ and the dosages of Ti-KBC 2.5 g·L^−1^, as the number of cycles increased, the degradation rate of enrofloxacin gradually decreased from 84.63% to 77.14%, which was still higher than that of BC, Ti-BC and Ti-FBC.

## Discussion

In order to study the degradation mechanism of enrofloxacin by BC, Ti-BC, Ti-FBC and Ti-KBC, we used EDS, XRD, FT-IR, XPS, etc. to characterize the structure of four kinds of biochar. After modification, the surface of the biochar can adhere to more TiO_2_, so the Ti-FBC and Ti-KBC have a larger specific surface area, and t he stronger the degradation performance of pollutants for the modified biochar. That the loaded TiO_2_ was uniformly distributed on the surface and the pores of the composite(biochar) not only provided the sufficient sites for adsorbing enrofloxacin, but also had a good photocatalytic activity for the degradation of enrofloxacin. By compared the unmodified biochar, after the iron modification and alkalization modification, the more TiO_2_ can be supported on the surface of the biochar, so the degradation of enrofloxacin were better.

In the photocatalytic degradation reaction, the smaller the band gap of the material, the easier it is to be excited by visible light. The more photo-generated electrons in the valence band transition to the conduction band, the more holes in the valence band participate in the oxidation reaction, and the degradation efficiency higher^23^. The UV-vis DRS analysis shows that Ti-KBC has the lowest band gap, so the slower the recombination rate of photogenerated electron-hole pairs, and the photocatalytic activity of the Ti-KBC was improved, which was consistent with the results measured by the photodegradation test.

TiO_2_ supported on the surface of Ti-BC, Ti-FBC and Ti-KBC generated the electron-hole pair and reacted in the solution system to generate strong oxidizing active groups such as hydroxyl radical (•OH) and superoxide ion radical (O_2_•^—^)^24^ under the excitation of ultraviolet light, therefore, the degradation rate of enrofloxacin by TiO_2_-loaded biochar improved effectively. Compared with unmodified biochar, the modified biochar had more abundant specific surface area and pore structure, and could attach more TiO_2_, consequently, the lifetime of photogenerated electrons increased and the photodegradation efficiency improved significantly. In addition, the basic oxygen-containing groups on the surface of the alkalized modified biochar increased greatly, and these oxygen-containing groups acted on the nano-TiO_2_ particles^25^, which made Ti-KBC had the most efficient degradation of enrofloxacin.

Enrofloxacin, being an ionic organic compound with a dissociation constant of pK_a1_ = 6.02 and pK_a2_ = 8.25^26^, exists in different ionic forms at different pH. Since the surface of Ti-BC, Ti-FBC and Ti-KBC were negatively charged, this favors enrofloxacin adsorption in the cationic form. When the pH < 5.0, the adsorption of enrofloxacin on the biochar reduced obviously because a large amount of H^+^ in the solution competed with the cation form of enrofloxacin. At the pH range of 5.0–6.02, it was the pH environment in which biochar had the best adsorption effect on enrofloxacin, and further the composite material had the best degradation effect on enrofloxacin. At pH > 6.02, enrofloxacin was mainly anionic in solution, which was not conducive to adsorption by negatively charged BC, Ti-BC, Ti-FBC, and Ti-KBC on the surface, leading to a decrease in the amount of adsorption, which limited the enrofloxacin degradation. When the biochar was added in a certain amount, the active substances produced in the solution system were limited. As the initial concentration of enrofloxacin in the solution increased, the produced active substances could not degrade enough enrofloxacin, thus leading to the degradation rate of enrofloxacin gradually decreased.

Due to the photocatalytic effect of TiO_2_, enrofloxacin adsorbed on Ti-KBC was degraded into small molecules under the irradiation of ultraviolet light, the re-adsorption capacity of Ti-KBC was enhanced, and the reuse of Ti-KBC performance was also improved. It was resulted in the higher the degradation rate of Ti-KBC for enrofloxacin through five cycles of tests.

## Conclusions

In the study, the TiO_2_-modified biochar was synthesized by impregnation and calcination. The SEM, EDS, FT-IR, XRD, XPS analysis showed that TiO_2_ was loaded on the composite material in anatase state. Through UV-vis DRS analysis of biochar, the band gaps of Ti-FBC and Ti-KBC were lower and have higher photocatalytic activity. Under 15 W UV lamp (254 nm) irradiation, the photodegradation reaction of the unmodified biochar(BC), TiO_2_-biochar(Ti-BC), TiO_2_-ironized biochar(Ti-FBC) and TiO_2_-alkaline biochar(Ti-KBC) for enrofloxacin conformed to the first-order kinetic model. After 60 minutes of UV irradiation, the maximum degradation effect was achieved and the Ti-KBC showed the optimum degradation effect of enrofloxacin. At the solution pH 5.0 and the dosage of Ti-KBC 2.5 g·L^−1^, the degradation rate of 100 mg·L^−1^ enrofloxacin reached 85.25%. After five cycles of tests, the degradation rate of Ti-KBC for enrofloxacin solution reached 77.14%, which indicated that the Ti-KBC was more feasible to degrade enrofloxacin.

## Materials and Methods

The test primary biochar was made from corn stalk through a 20 mesh sieve, pyrolyzed in a muffle furnace at 773 K for 3 h, and after pyrolysis, cooled to room temperature, and passed through a 60 mesh sieve. The sample was marked as BC.

### Synthesis of TiO_2_-biochar composite

First, takeing 25 ml of absolute ethanol into a beaker, and adding 2 ml of butyl titanate slowly under stirring with a magnetic stirrer, thorough mixing, and then adding 1 ml of distilled water, glacial acetic acid and nitric acid,repectively, through mixing again, and continue to add 1 ml of distilled water, glacial acetic acid and nitric acid, repectively, stirring for 30 min and finally adding 5 g of biochar, contnue to stir for 30 min, the mixture obtained was dried in an oven at 378 K, andthen, calcined at 773 K for 3 h in a muffle furnace, and finally cooled to room temperature to obtain a composite material (marked as Ti-BC). Secondly, BC(10.0 g) was added to 100 mL of 0.1 mol·L^−1^ Fe^3+^ solution which was fully soaked at a ratio of 1:10, and placedon a constant temperature agitator, oscillated every 8 h and aged for 24 h. After the reaction was completed, it is washed repeatedly with distilled water until neutral, placed in an oven (378 K) for 24 h. After modification, the operations of loading TiO_2_ were the same as Ti-BC, the sample was marked as Ti-FBC. Thirdly, BC(10.0 g) was added to 100 ml of 25% KOH solution and fully soaked at a 1:10 impregnation ratio. Other operations were the same as Ti-FBC, the sample was marked as Ti-KBC.

### Photocatalytic performance test

Weigh 0.5000 g of BC, Ti-BC, Ti-FBC and Ti-KBC into a 250 ml beaker and add 200 ml of enrofloxacin solution with an initial concentration of 100 mg·L^−1^. At 298 K, the temperature was kept away from light for 12 h. After reached the adsorption-desorption equilibrium, the photodegradation test was performed under the irradiation of ultraviolet light (254 nm) generated by a 15 W ultraviolet lamp. When the degradation times were 0, 15, 30, 45, 60, 120, 180, 240, 300, 360 min, sample the solution separately. The supernatant was passed through a 0.45 μm membrane, and the content of enrofloxacin in the solution was determined by a liquid chromatograph. The initial enrofloxacin concentration was varied to 20, 50, 100, 150, 200 mg·L^−1^, to explore the effects of different initial concentrations on photodegradation. Adjust the initial pH of the solution to 3.0, 5.0, 7.0, 9.0, 11.0, to explore the effects of different initial pH on photodegradation. Changing the light source of photodegradation and to explore the degradation characteristics in both UV(15 W) and visible light(15 W) conditions. All the above experiments were performed with 3 groups of duplicates, and the data were fitted and analyzed using origin 8.5.

### Active substance detection

Weigh four 0.5000 g Ti-KBC into a 250 ml beaker and add 200 ml of enrofloxacin solution with an initial concentration of 100 mg·L^−1^. One directly subjected to photocatalytic degradation test, the other three were added with p-benzoquinone (O_2_•^—^ quencher^27^, isopropanol (•OH quencher^28^, potassium iodide (•OH and h^+^ quencher^29^. After the dark reaction is over, the photocatalytic test is started. The above tests were all set to 3 groups of repetitions.

### Cycle test of TiO_2_-modified biochar

Ti-KBC was used as the sample to perform the cycle test. Weigh 0.5000 g of Ti-KBC into a 250 ml beaker and add 200 ml of enrofloxacin solution with an initial concentration of 100 mg·L^−1^, initial pH = 5.0. After the adsorption-degradation reaction ended, filter out the remaining liquid and add 100 mg·L^−1^ again of enrofloxacin solution and continue the reaction. Setting up 5 times cycle tests according the above test step.

### Characterization of biochar

The surface acidic and basic group content of biochar is determined according to the Boehm method^30^; Specific surface area and pore structure of biochar was determined by specific surface area and pore size analyzer^31^; Microscopic characterization and EDS analysis of biochar using scanning electron microscope (SSX-550)^32^; The distribution of functional groups on the surface of biochar was analyzed by infrared spectrometer(FTIR)^33^; The crystal structure of biochar was determined by X-ray diffraction spectroscopy (XRD) and X-ray photoelectron spectroscopy (XPS)^33^; The optical properties of the composites were measured by a UV-visible spectrum scanner(UV-vis DRS).

### Determination of enrofloxacin

Chromatographic conditions: column (ZORBAX Eclipse XDB-C18 150 mm × 4.6 mm); Column temperature 303 K; injection volume 20 μL; Determination of enrofloxacin mobile phase was methanol: water = 30:70 (V/V) The flow rate was 1.0 mL·L^−1^, the UV detection wavelength was 275 nm, and the retention time was 4.6 min.
